# Comparative Analysis of Thymic and Blood Treg in Myasthenia Gravis: Thymic Epithelial Cells Contribute to Thymic Immunoregulatory Defects

**DOI:** 10.3389/fimmu.2020.00782

**Published:** 2020-05-06

**Authors:** Frédérique Truffault, Dani Nazzal, Julien Verdier, Angeline Gradolatto, Elie Fadel, Régine Roussin, Bruno Eymard, Rozen Le Panse, Sonia Berrih-Aknin

**Affiliations:** ^1^Sorbonne Université, INSERM, Institut de Myologie, Centre de Recherche en Myologie, Paris, France; ^2^Marie Lannelongue Hospital, Le Plessis-Robinson, France; ^3^AP-HP, Referral Center for Neuromuscular Disorders, Pitié-Salpêtrière Hospital, Institute of Myology, Paris, France

**Keywords:** myasthenia gravis, thymus, PBMC, thymic epithelial cells, Treg, CD31, TSLP, immune regulation

## Abstract

The thymus is involved in autoimmune Myasthenia gravis (MG) associated with anti-acetylcholine (AChR) antibodies. In MG, thymic regulatory T cells (Treg) are not efficiently suppressive, and conventional T cells (Tconv) are resistant to suppression. To better understand the specific role of the thymus in MG, we compared the phenotype and function of peripheral and thymic Treg and Tconv from controls and MG patients. Suppression assays with thymic or peripheral CD4 + T cells showed that the functional impairment in MG was more pronounced in the thymus than in the periphery. Phenotypic analysis of Treg showed a significant reduction of resting and effector Treg in the thymus but not in the periphery of MG patients. CD31, a marker lost with excessive immunoreactivity, was significantly reduced in thymic but not blood resting Treg. These results suggest that an altered thymic environment may explain Treg differences between MG patients and controls. Since thymic epithelial cells (TECs) play a major role in the generation of Treg, we co-cultured healthy thymic CD4 + T cells with control or MG TECs and tested their suppressive function. Co-culture with MG TECs consistently hampers regulatory activity, as compared with control TECs, suggesting that MG TECs contribute to the immune regulation defects of MG CD4 + T cells. MG TECs produced significantly higher thymic stromal lymphopoietin (TSLP) than control TECs, and a neutralizing anti-TSLP antibody partially restored the suppressive capacity of Treg derived from co-cultures with MG TECs, suggesting that TSLP contributed to the defect of thymic Treg in MG patients. Finally, a co-culture of MG CD4 + T cells with control TECs restored numbers and function of MG Treg, demonstrating that a favorable environment could correct the immune regulation defects of T cells in MG. Altogether, our data suggest that the severe defect of thymic Treg is at least partially due to MG TECs that overproduce TSLP. The Treg defects could be corrected by replacing dysfunctional TECs by healthy TECs. These findings highlight the role of the tissue environment on the immune regulation.

## Introduction

Myasthenia gravis (MG) is a chronic autoimmune disorder caused, in most patients, by anti-acetylcholine receptors (AChR) antibodies, which mainly destroy AChR at the neuromuscular junction, leading to muscle weakness and fatigability (1). Accumulating arguments strongly support that the thymus plays a role in the pathology of MG (2). Indeed, thymectomy has favorable clinical effects, especially in young patients (3). In addition, functional and morphological abnormalities of the thymus occur very frequently in MG patients: about 50% of them present thymus hyperplasia with the development of lymphoid follicles, and 10 to 15% have an epithelial tumor of the thymus (4). Thymic hyperplasia is particularly common in young women with a high level of anti-AChR antibodies (5), that decreases after thymectomy in association with clinical improvement (6). Thus the thymus seems to play a key role in anti-AChR antibody production.

Several signs of activation and inflammation can be found in the hyperplastic thymus of MG patients. B cells spontaneously produce anti-AChR antibodies (7), and CD4 + T cells express higher levels of Fas/CD95 (8) and proliferate more in response to recombinant interleukin (IL)-2 than CD4 + T cells from control individuals (9). In addition, suppression by CD4 + CD25 + thymic regulatory T cells (Treg) is severely reduced in MG patients compared with controls (10), and CD4 + CD25- thymic conventional cells (Tconv) exhibit resistance to the suppressive activity (11). Taken together, these findings suggest that the thymus is chronically activated in MG.

Using microarray experiments, we showed that type I- and type II-interferon (IFN)-regulated genes are highly expressed in MG thymus in comparison with control thymus (12). Although the origin of the thymic inflammation is still unclear, we showed that molecules mimicking a viral infection increase the expression of the autoantigen (AChR) as well as that of chemokines (13) through the production of IFN-β that appears to play a central role in MG thymic changes (14).

Despite evidence of a dysregulated immune response in the thymus of MG patients, the role of thymic epithelial cells (TECs) has been only scarcely considered. It was shown that TECs from MG patients overproduce IL-1, IL-6, and RANTES in comparison with TECs from healthy subjects (9, 15). Recently, we showed that the overproduction of IL-17 in MG thymuses is sustained by a higher secretion of IL-23 by MG TECs (16).

In control thymuses, medullary TECs (mTECs) promote the generation of thymic Treg and favor their function, an effect mainly due to IL-2 overproduction by thymic CD4 + conventional T cells (Tconv) (17). Treg were first defined by their high expression of CD25 (18, 19), then by the expression of FoxP3, a master control gene for Treg development and function (20). In humans, however, FoxP3 is not exclusively expressed in Treg as it can be transiently induced in TCR-stimulated naïve CD4 + T cells without conferring any suppressive activity (21). The absence of CD127 is also a hallmark of Treg, yet some CD25hiCD127low CD4 + T cells also contain non-Treg (22, 23). In addition, the combination of FoxP3 and CD45RA can separate three phenotypically and functionally distinct subpopulations: CD45RA + FoxP3lo resting Treg (rTreg) and CD45RA-FoxP3hi effector Treg (eTreg) that are both suppressive *in vitro*, while cytokine-secreting CD45RA-FoxP3lo cells (Fraction III, FIII) are non-suppressive (24). Finally, the co-expression of chemokine receptors in Treg identifies specific functional subsets. Treg expressing chemokine receptors travel to the sites of inflammation to deliver a suppressive activity. As an example, CD4 + FoxP3 + CXCR5 + cells defined as follicular regulatory T cells control follicular effector T cells (CXCR5 + FoxP3neg) in lymphoid follicles (25).

In order to better understand the specific role of the thymus in MG, we compared peripheral and thymic Tconv and Treg from controls and MG patients by combinations of markers, and we addressed whether TECs from control and MG patients had distinct immune imprinting properties. We showed that the phenotypic and functional defects in MG were more pronounced in the thymus than in the periphery and that TECs from MG patients induced immune regulation defects in control CD4 + T cells, thus demonstrating that mTECs in MG patients contribute to the immunoregulatory defects in MG.

## Materials and Methods

### Thymuses and Blood Samples

Thymuses were obtained from MG patients (12–41 year-old) undergoing thymectomy at the “Hôpital Marie Lannelongue” or “Centre Hospitalier Universitaire de Strasbourg.” The clinical details of the 44 patients included in the study are described in Table 1. Normal thymuses were obtained from infants (4 days to 11 years) and adults (13–35 years) undergoing cardiac surgery at the “Hôpital Marie Lannelongue.”

**TABLE 1:** Clinical characteristics of MG patients included in the study.

Age (median)	29 years	
Sex	39 (89%)	Female
	5 (11%)	Male
Antibody status	40 (91)	Anti-AChR positive
	4 (9%)	Seronegative
	1 (2%)	Anti-MuSK positive
Therapy at time of analysis	43 (98%)	Acetylcholinesterase inhibitors
	2 (5%)	Plus corticosteroids
	1 (2%)	Plus immunosuppression
MG clinical subtype	31 (70%)	Generalized MG
	12 (27%)	Ocular MG
	1 (2%)	Not defined
Thymectomy	41 (93%)	Yes
	3 (7%)	No
Thymic histology	31 (70%)	Hyperplasia
	1 (2%)	Thymoma
	7 (18%)	No abnormality detected
	2 (5%)	Not known

Thymocytes were isolated from thymuses by mechanical dissociation of fresh thymic tissue, as previously described (26). The cells were filtered through cell strainer device to remove thymic tissues and washed once with HBSS.

Blood was obtained from MG patients (19–45 years old) just before thymectomy or during follow–up consultation, and from sex-matched control donors (20–64 years old) from the “Etablissement Français du Sang” (EFS). Peripheral blood mononuclear cells (PBMCs) were isolated by Ficoll density gradient centrifugation (Eurobio, Les Ulis, France).

These investigations were approved by the local Ethics Committee (“Comité Consultatif de Protection des Personnes”), Ile de France VII (Kremlin Bicêtre, France). The relevant authorization numbers are ID RCB 2006-A00164-47 and 2010-A00250-39.

### Isolation of CD4+ T Cells

CD4 + T cells were obtained from fresh thymic tissue or from fresh PBMCs, as previously described (10). Total CD4 +, CD4 + CD25 +, or CD4 + CD25- cells were purified using magnetic separation according to the manufacturer’s instructions (Dynabeads CD4 + CD25 + Treg Separation Kit, Life Technologies, Saint Aubin, France and CD4 + CD25 + regulatory T cell Isolation Kit, Miltenyi, Paris, France). For the isolation of CD4 + thymic cells, we added an anti-CD8 antibody (AbSerotec, Düsseldorf, Germany) to eliminate double-positive cells expressing both CD4 and CD8.

### Flow Cytometry

Staining was performed on fresh total and frozen CD4 + thymocytes, as well as on frozen PBMCs. The antibodies used in this study are described in Table 2. Two different combinations of antibodies were used and are described in Table 3. Data were acquired on a BD FACS CALIBUR then analyzed using Cellquest software (United States) for the combination of 4 colors, and on BD FACS Verse [with FACSuite software then analyzed using FlowJo Software (United States)] for the combination of 6 colors. Gating strategies to identify subpopulation of Treg are shown in Supplementary Figure 1.

**TABLE 2:** List of antibodies used in cytometry experiments.

Specificity	Label	Type	Clone	Producer
CD4	FITC	Mouse IgG1, κ	MT 310	DAKO
CD4	PE	Mouse IgG1, κ	MT 310	DAKO
CD4	APC	Mouse IgG1, κ	MT 310	DAKO
CD4	PerCp-Cy5.5	Mouse IgG1, κ	RPA-T4	BECTON DICKINSON
CD8	APC	Mouse IgG1, κ	DK25	DAKO
CD8	PE-Cy7	Mouse IgG1, κ	RPA-T8	BECTON DICKINSON
CD25	PE-Cy7	Mouse IgG1, κ	2A3	BECTON DICKINSON
CD25	PE	Mouse IgG1, κ	ACT-1	DAKO
CD31	PerCp-eFluor 710	Mouse IgG1, κ	WM-59	eBioscience
CD45RA	FITC	Mouse IgG2b	H1100	BECTON DICKINSON
CD95	PE	Mouse IgG1, κ	DX2	BECTON DICKINSON
CD95	APC	Mouse IgG1, κ	DX2	BECTON DICKINSON
CD127	Brilliant Violet 421	Mouse IgG1, κ	HIL-7R-M21	BECTON DICKINSON
CXCR3	FITC	Mouse IgG1	49801	R&D Systems
CCR4	PE	Mouse IgG2b	205410	R&D Systems
CCR4	FITC	Mouse IgG2b	205410	R&D Systems
CXCR5	Biotin	Mouse IgG2b	51505	R&D Systems
CXCR5	Alexa Fluor 488	Rat IgG2b	RF8B2	R&D Systems
CCR7	FITC	Mouse IgG2a	150503	R&D Systems
CCr7	APC	Mouse IgG2a	150503	R&D Systems
Foxp3	eFluor 660	Mouse IgG1, κ	236A/E7	eBiosciences

**TABLE 3:** Combination of antibodies used in flow cytometry experiments.

A. Combinations of 4 antibodies			
Antibody 1	Antibody 2	Antibody 3	Antibody 4
CD4 FITC	CD95 PE	CD25 PE-Cy7	CD8 APC
CD4 FITC	CCR4 PE	CD25 PE-Cy7	CD8 APC
CD4 FITC	CD25 PE	CXCR5 biotin + Streptavidin PE-CY7	CD8 APC
CXCR3 FITC	CD4 PE	CD25 PE-Cy7	CD8 APC
CCR7 FITC	CD4 PE	CD25 PE-Cy7	CD8 APC

B. Combinations of 5 or 6 antibodies					
Antibody 1	Antibody 2	Antibody 3	Antibody 4	Antibody 5	Antibody 6
CD4 APC	CD8 PE-Cy7	CD25 PE	CD127 BV 421	CD45RA FITC	CD31 PerCp-eF 710
CD4 PerCp-Cy5.5	CD8 PE-Cy7	CD25 PE	CD127 BV 421	CD45RA FITC	FOXP3 eF660
CD4 PerCp-Cy5.5	CD8 PE-Cy7	CD25 PE	CD127 BV 421	CXCR3 FITC	
CD4 PerCp-Cy5.5	CD8 PE-Cy7	CD25 PE	CD127 BV 421	CXCR5 Alexa Fluor 488	
CD4 PerCp-Cy5.5	CD8 PE-Cy7	CD25 PE	CD127 BV 421	CCR4 FITC	CCR7 APC
CD4 PerCp-Cy5.5	CD8 PE-Cy7	CD25 PE	CD127 BV 421	CD95 APC	

### Cell Culture

Cell culture products (Hank’s balanced salt solution (HBSS), RPMI 1640 Glutamax I medium, Penicillin, Streptomycin, Trypsin) were obtained from Invitrogen (Cergy-Pontoise, France) while sera were obtained from Eurobio (Les Ulis, France) and Ultroser-G from PALL-Biosepra (Cergy-Pontoise, France).

Primary TEC cultures were obtained from mechanically minced fresh human thymus tissue and seeded onto cell culture flasks, as previously detailed (27). These culture conditions result mainly in medullary TECs (mTECS) that remain functional (27). Indeed, mTECs in primo-cultures keep their ability to express key molecules involved in immune tolerance processes such as autoimmune regulator, tissue-specific antigens, chemokines, and cytokines (27). After 8 to 12 days of primary culture, the confluent monolayers were trypsinized and used in co-culture experiments, or frozen for further use.

### Cocultures

Freshly purified CD4 + thymocytes (5 × 10^5^ cells/well) were cultured alone, or cocultured with mTECs, in 24-well plates in RPMI 1640 Glutamax I medium supplemented with 10% fetal calf serum as previously described (17). For staining experiments, CD4 + cells were cocultured with 2 × 10^5^ mTECs during 24 h. For immunosuppression assay, the CD4 + were cocultured with 1 × 10^5^ mTECs for 3 days.

### Suppressive Assay

The suppressive activity of CD4 + T cells cultured alone or with mTECs was evaluated by tritiated thymidine incorporation, as previously described (10). The suppressive capacity of Treg was normalized as the percent of proliferative response of phytohaemagglutinin-activated Tconv alone. In some experiments, blocking anti-TSLP (R&D Systems, Lille, France) antibody was used at 0.1 μg/ml.

### Enzyme-Linked Immunosorbent Assay

Thymic stromal lymphopoietin (TSLP) concentrations were measured in duplicate from supernatants of cultures of TECs, using commercially available enzyme-linked immunosorbent assays (Peprotech, France) according to the manufacturer’s instructions. Measurements were performed on 100 μl of cell culture supernatant previously frozen and analyzed on a MRX Revelation microplate reader from Dynex (Thermolab System, Chantilly, VA, United States).

### Statistical Analyses

Differences between groups were evaluated using parametric or non-parametric *t*-tests for paired or unpaired data (Graph Pad Software, San Diego, CA, United States), with the significance level set to *p* < 0.05. Each figure legend mentions the statistical test used. Other statistical tests have been used and are indicated in the text (One-way anova to compare 3 groups and Spearman non-parametric correlation).

## Results

### Functional Characterization of Thymic and Peripheral Regulatory Cells

The suppression function of Treg is profoundly impaired in the MG thymus (10). In order to investigate whether peripheral Treg behave similarly, we compared the suppressive function of Treg isolated from the thymus or from peripheral blood cells from MG and control donors.

**FIGURE 1:**
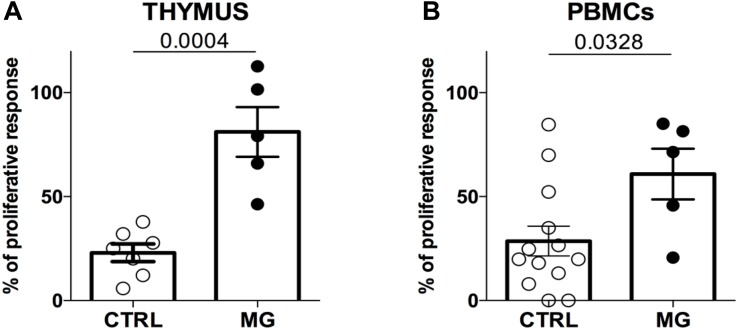
The suppression function is more impaired in the thymus than in the periphery in MG patients. Percentages of proliferation of Tconv in co-culture with Treg (ratio 1:1) from control individuals (CTRL) or patients with myasthenia gravis (MG) using cells derived from the thymus **(A)** or from PBMCs **(B)**. Data represent the mean ± standard error of the mean. Statistical test: Two-tailed *t*-test.

In both compartments, the suppressive function was impaired in MG patients. In the thymus, the average proliferation was 23.0% for controls and 81.1% for MG patients (Figure 1A, *p* < 0.001). In PBMCs, the average proliferation was 29.4% for controls and 60.8% for MG patients (Figure 1B, *p* < 0.04). While in the thymus, there was no overlap between MG and control values, it was not the case for PBMC results (Figures 1A,B).

The more impaired suppressive activity of thymic Treg compared to peripheral Treg suggests that Treg from these two compartments might present phenotypic differences.

### Phenotypic Characterization of Thymic and Peripheral Regulatory Cells

To analyze the phenotype of Treg, we used several combinations of markers. We first analyzed the level of CD25 expression. We then investigated the combination of CD25 with CD127 since CD25 + CD127neg cells are defined as regulatory cells (22). Finally, to further define most precisely the subtypes of Treg, we used the CD45RA marker together with CD25 and FoxP3 to differentiate resting (CD45RApos CD25lo FoxP3lo) (rTReg), effector Treg (CD45RAneg CD25hi FoxP3hi) (eTReg) and CD45RAneg CD25lo FoxP3lo (CD25lo non-Treg, defined as FIII) cells as previously described (24). The gating strategy is shown in Supplementary Figure 1.

**FIGURE 2:**
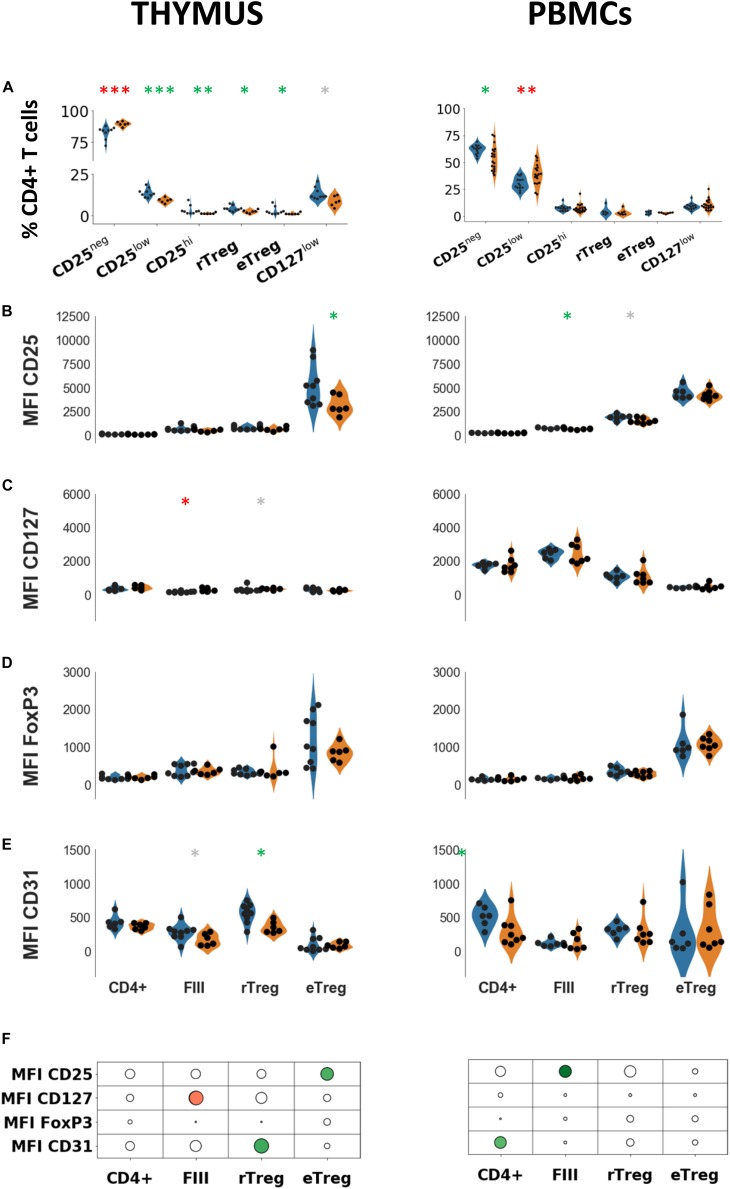
Phenotypic characterization of thymic and peripheral regulatory cells. **(A)** Percentages of indicated cell populations among total CD4 + T cells in healthy individuals (blue) or in myasthenic patients (orange) in the thymus (left panel) or in PBMCs (right panel). Mean fluorescent intensities of CD25 **(B)**, FoxP3 **(C)**, CD127 **(D)**, CD31 **(E)** in healthy individuals (blue) or in myasthenic patients (orange) in the 3 subsets defined by the combination of CD45RA and CD25 in thymus (left panel) or in PBMCs (right panel). Statistical test: two-tailed Mann-Whitney (^∗^*p* ≤ 0.05; ^∗∗^*p* ≤ 0.01; and ^∗∗∗^*p* ≤ 0.005, gray star corresponds to *p* < 0.1). **(F)** Statistical summary of the data shown in **(B–E)**. Colorized bubbles correspond to significant differences. Size is proportional to the statistical significance and color represents fold change between mean values of control individuals and MG patients (green, lower value in MG; red, higher value in MG).

In MG patients, the percentages of Treg were reduced in the thymus but not in the periphery regardless of the gating strategy to define Treg (Figure 2A). Fractioning Treg subsets based on CD45RA expression revealed that percentages of both resting and effector Treg were reduced in the thymus of MG patients but not in the periphery (Figure 2A). As a consequence of Treg reduction, the percentage of CD25neg cells was increased in the thymus (Figure 2A, left panel). In PBMCs, the percentage of CD25neg was decreased together with an increase in CD25lo cells (Figure 2A, right panel). These results show a very dissimilar phenotype of Treg in the thymus and PBMCs.

To characterize more precisely the phenotypic changes of the subsets defined with CD45RA (rTReg, eTreg FIII) in the thymus and PBMCs, we determined mean fluorescent intensities of markers associated with Treg function. In the thymus of MG patients, CD25 expression was significantly reduced in eTreg but not in the other subsets while in PBMCs a decrease in CD25 expression was observed in FIII (Figure 2B) but not in the other subsets. The level of CD127 was higher in FIII (*p* < 0.02) and in rTreg without reaching statistical significance (*p* = 0.066) in the MG thymus but not in PBMCs (Figure 2C).

CD127 expression is expected to be low in the two Treg subsets (rTreg and eTreg) and high in FIII. This was the case in the periphery (*p* < 0.001, one-way ANOVA for controls and MG) where an inverse correlation between CD25 and CD127 was observed (*r*^2^ = −0.77 for MG patients and *r*^2^ = 0.87 for controls, *p* < 0.0001, Spearman correlation test), but not in the thymus. CD127 expression was much lower in the thymus as compared with PBMCs (4 to 5 fold, *p* < 0.0001 for both MG and control groups, *t*-test) and the level in the thymus was similar in the different subpopulations (One-way ANOVA test).

As expected, the level of FoxP3 was higher in eTreg compared to the other subsets in both thymus and PBMCs (Figure 2D), but there was no change at all in MG patients compared to controls, both in the thymus and in PBMCs (Figure 2D).

We also investigated the expression of CD31 (PECAM-1), a marker of recent thymic emigrants that is lost with excessive immunoreactivity (28). The expression of CD31 in thymic CD4 + cells from MG patients was significantly reduced in rTreg while it was unchanged in eTreg, and a trend for a decrease in FIII was observed (Figure 2E, left panel). In PBMCs, the level of CD31 was unchanged in MG in the three different subsets although a decrease in CD31 expression was observed in the whole CD4 population (Figure 2E, right panel). A decrease in CD31 is consistent with an increased proliferation and activation of these cells (29). CD31 expression was reported to be higher in circulating FoxP3 + rTreg than in eTreg (30) (albeit in the absence of statistical testing), we observed this profile more clearly in the thymus than in the blood.

The results are summarized in the Figure 2F showing that the alterations of CD4 cells are dissimilar in the thymus and PBMCs. In the thymus, alterations were observed in all cell subsets, while in PBMCs, Treg subsets (rTreg and eTreg) did not appear to be affected.

### Expression of CD95 and Chemokine Receptors in Treg Subsets

**FIGURE 3:**
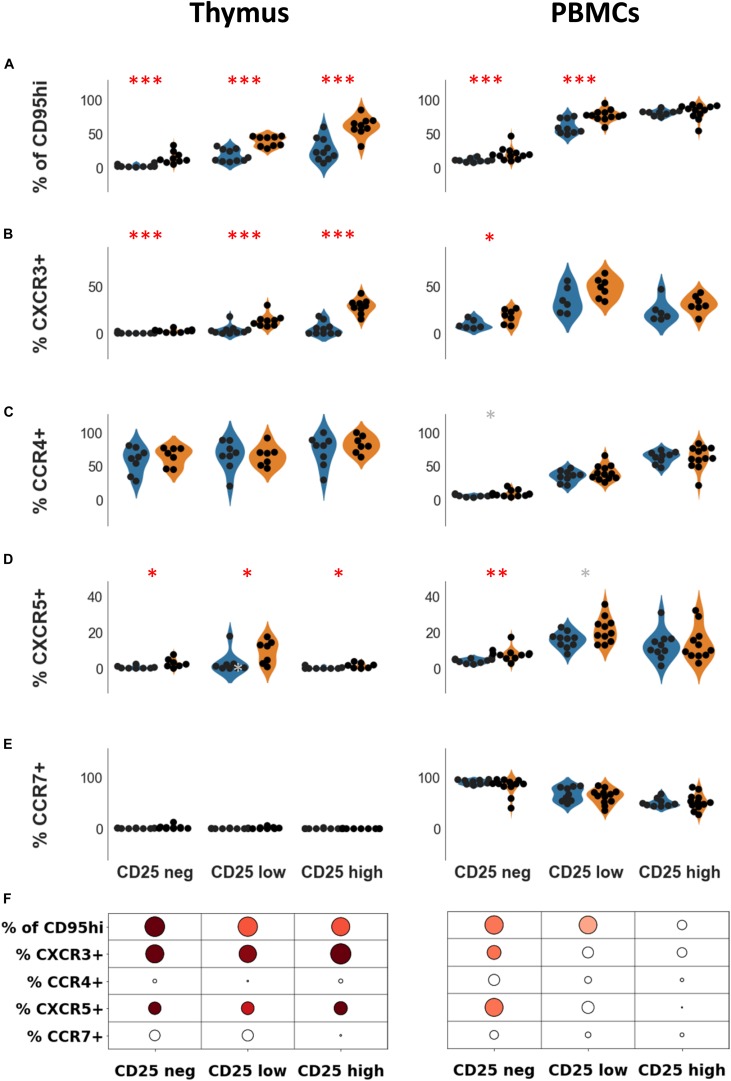
Expression of CD95 and chemokine receptors in Treg subsets. Percentages of CD95hi **(A)** CXCR3 **(B)**, CCR4 **(C)**, CXCR5 **(D)**, and CCR7 **(E)** according to CD25 expression, in healthy individuals (blue) or in myasthenic patients (orange) in the thymus (left panel) or in PBMCs (right panel). Statistical test: two-tailed Mann-Whitney (^∗^*p* ≤ 0.05; ^∗∗^*p* ≤ 0.01; and ^∗∗∗^*p* ≤ 0.005, gray star corresponds to *p* < 0.1). **(F)** Statistical summary of the data shown in this figure. Colorized bubbles correspond to significant differences. Size is proportional to the statistical significance and color represents fold change between mean values of control individuals and MG patients (green, lower value in MG; red, higher value in MG).

In healthy subjects, the percentage of CD95 + cells increased with the level of CD25 on CD4 + T cells but more moderately in the thymus (*p* = 0.054, Spearman correlation test) than in the periphery (*p* < 0.0001, Spearman correlation test) (Figure 3A). Confirming what we previously showed (10), the percentage of CD95 + cells was significantly higher in thymocytes from MG patients compared with controls in all subpopulations (CD25neg, CD25lo, and CD25hi) (Figure 3A, left panel). In PBMCs, higher percentages of CD95 + cells were also observed in CD25neg and CD25low populations, but not in the CD25hi cells (Figure 3A, right panel).

Trafficking receptors expressed on Treg undergo changes according to their stages of activation and differentiation (31). CXCR3 and CXCR5 levels are increased in CD4 + T cells in the thymus and PBMCs of MG patients (32, 33). The thymic overexpression of CXCR3 and CXCR5 on CD4 + T cells in MG patients was independent of CD25 expression (Figures 3B,D, left panel). In PBMCs, the overexpression of CXCR3 and CXCR5 on CD4 + T cells was observed in CD25neg and CD25lo cells but not in CD25hi cells (Figures 3B,D, right panel).

In the thymus, the levels of CCR4 were high (> 60% in all CD4 + T cell subsets) (Figure 3C, left panel) while CCR7 was hardly detectable (Figure 3E, left panel). In PBMCs, the level of CD25 expression on CD4 + T cells was correlated with the level of CCR4 (*p* < 0.0001, Spearman correlation test) and inversely correlated with the level of CCR7 (*p* < 0.001, Spearman correlation test). Nevertheless, levels of CCR7 and CCR4 on CD4 + T cells were unaltered in MG both in the thymus (Figures 3C,E, left panel), and in PBMCs (Figures 3C,E, right panel).

Altogether, immune dysregulations were more pronounced in the thymus than periphery although some modifications were also found in the periphery such as CD95 and chemokine receptors (Figure 3F). Changes in the CD25hi subset was only observed in the thymus. These results suggest that the altered thymic environment in MG may directly contribute to the phenotypic differences we observed between MG patients and controls.

### Association Between Immune Defects and Clinical Features

To address whether clinical aspects of the disease affected the observed differences, we confronted the results shown in Figures 2, 3 with available clinical data. Although some analyses were not conclusive due to a low number of samples, several interesting observations were made.

#### Thymus Pathology

We observed that the expression of several markers was positively correlated with the germinal center grade in the thymus: CD95 in CD25hi (*p* < 0.05) and CXCR3 in CD25lo (*p* < 0.005), suggesting that the changes in thymic cell subsets involve CD25 positive cells (including activated and regulatory cells) (Supplementary Figures 2A,B). Besides, we observed some changes in PBMCs: the percentage of CD25 + CD127- cells increase with the thymic germinal center grade (Supplementary Figure 2C) suggesting that thymic events could impact Treg changes in the periphery.

#### Duration of the Disease

We did not observe any correlation between the duration of the disease and any biological parameter in the thymus, but the number of samples in each subgroup was small. In PBMCs, we found that patients with a long disease duration tended to have higher expression of CD25 and a higher percentage of CD25loCD45RAneg (FIII) (Supplementary Figures 2D,E).

#### Antibodies

Because the number of seronegative patients was low, it was not possible to determine whether the absence of auto-antibodies was related to the measured parameters. However, we did not observe any convincing correlation between measured parameters and levels of anti-AChR antibodies.

#### Clinical Grade

We observed interesting correlations between the clinical grade and Tconv/Treg balance in PBMCs. Percentages of CD25 + Treg and of CD25lo were higher in severely affected patients compared with mild patients, while it was the opposite for CD25neg Tconv cells (Supplementary Figures 3A–C). The percentage of CD25 + CD127- cells was also higher in patients with more severe disease (Figure 3D). Finally, the level of CCR4 in CD25lo cells was correlated with the clinical grade (Supplementary Figure 3E); the severely affected patients have a lower level of CCR4, which is a marker associated with immune regulation.

### TECs Contribute to the Functional Defect of Treg: Role of TSLP

We previously showed that the thymic epithelium plays a major role in the generation of Treg (17). We wondered whether regulatory function defects in MG could be explained by alterations of MG TECs.

**FIGURE 4:**
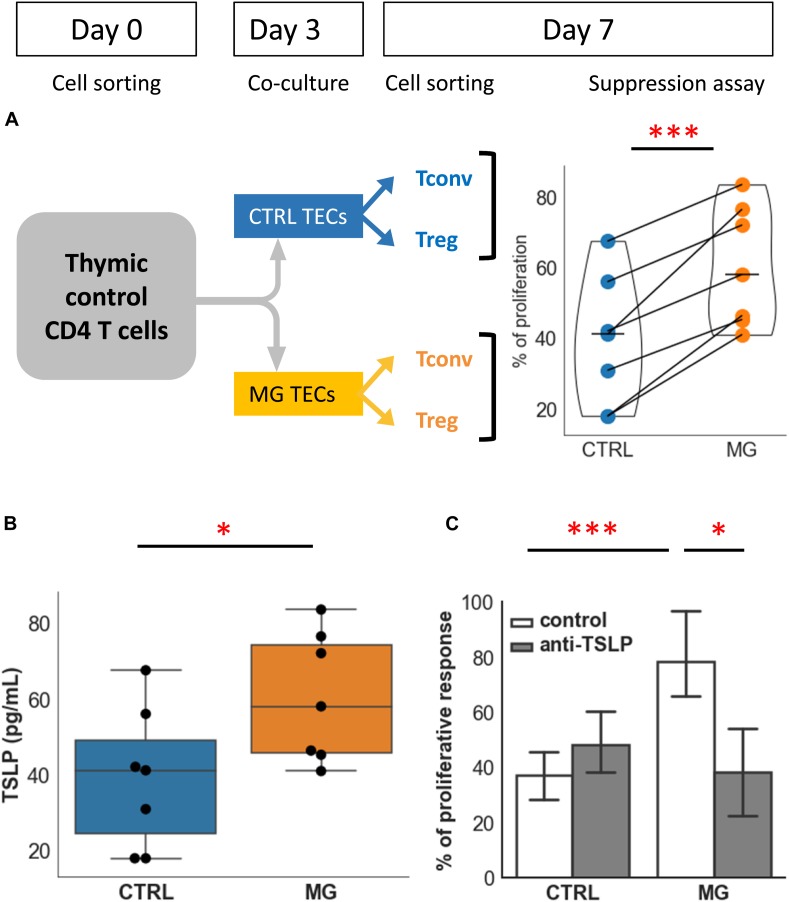
TECs contribute to the functional defect of Treg: role of TSLP. **(A)** Percentages of proliferation of T conv in co-culture with Treg following co-culture of CD4 + cells from control individuals with TECs either from control individuals (CTRL, blue) or from MG patients (MG, orange). The experimental protocol is explained in the top left panel. **(B)** Production of TSLP measured in supernatants of cultured TEC from control individuals (CTRL, blue) or MG patients (MG, orange). **(C)** Percentage of proliferation of Tconv in co-culture with Treg following co-culture with TEC from control individuals (CTRL) or MG patients as described in **(A)**, in the absence or presence of a TSLP neutralizing antibody. Statistical tests: two tailed paired *t*-test in **(A,C)**, two-tailed Mann-Whitney in **(B)** (^∗^*p* ≤ 0.05; ^∗∗∗^*p* ≤ 0.005).

To answer this question, we co-cultured healthy thymic CD4 + T cells with control or MG TECs for 3 days. Then Teff and Treg were purified, and we tested the suppressive function in a proliferation assay. We observed a consistent loss of regulatory activity in all experiments (*n* = 7) (Figure 4A). Indeed, co-culture with control TECs resulted in a mean percentage of proliferation that was 39% while co-culture with MG TECs resulted in a mean percentage of proliferation that was approximately 60%. The mean of differences between the 2 culture conditions was 21.3%. These results support the idea that MG TECs contribute to the immune regulation defects of MG T cells.

We then hypothesized that soluble factors produced by TECs and having an effect on Treg could mediate the pathogenic mechanism due to MG TECs. We investigated IL-6 and TSLP that are known factors influencing regulatory function (34, 35).

Supernatants produced by TECs from MG patients produced significantly higher levels of TSLP than that from controls (Figure 4B). In our co-culture experiments, a neutralizing anti-TSLP antibody partially restored the suppressive capacity of Treg derived from co-cultures with MG TECs (Figure 4C). This suggested that TSLP was one of the factors contributing to the defect of thymic Treg in MG patient. By contrast, anti-IL-6 antibody had no effect on the suppressive capacity of Treg (not shown).

To analyze whether the level of TSLP is also increased in the whole thymus, we performed real-time PCR in thymic samples, and observed a significant increase in TSLP in MG thymus compared with age-matched controls, although this global analysis does not allow to determine which cell type over produce TSLP (Supplementary Figure 4).

Altogether, these results show that TECs played a significant role in the defect of Treg in MG patients, and this effect was at least partially mediated by TSLP that was overproduced by MG TECs.

### Immune Regulation in MG CD4+ Cells Could Be Restored

**FIGURE 5:**
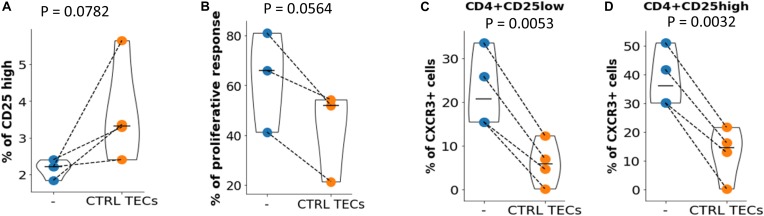
Immune regulation in MG CD4 + cells could be restored. **(A)** Percentages of CD25 high cells in CD4 + T cells from MG patients before (–) or after (CTRL TECs) co-culture with TEC from control individuals. **(B)** Proliferation of Tconv in co-culture with Treg before (–) or after (CTRL TECs) co-culture of thymic CD4 + cells from MG patients with TEC from control individuals (CTRL, blue). Percentage of CXCR3 + cells among CD4 + CD25low **(C)** or CD4 + CD25high **(D)** thymic CD4 + cells from MG patients before (–) or after (CTRL TECs) co-culture with TECs from control individuals. Statistical tests: two-tailed paired *t*-test.

The experiments described above indicated that thymic CD4 + T cells from MG patients present numerous defects affecting Treg. Since TECs appear to play a key role in these defects, we asked if it was possible to normalize the number and function of MG Treg by a coculture with control TECs. The coculture of thymic CD4 + T cells from MG patients in presence of control TECs induced an increase in the CD25high cell number in the 4 experiments (Figure 5A). To evaluate the suppressive function, CD4 + thymic MG cells were incubated with control TECs for 3 days and Treg and Tconv were purified for a suppression assay. The suppression defect was reduced in all experiments (3 different MG patients tested) (Figure 5B). Finally, normal TECs decreased the expression of CXCR3 in CD25lo and CD25hi cells (Figures 5C,D), but had no effect on CXCR5 (not shown).

Collectively, these results show that the immune regulation defects of T cells could be corrected when MG T cells were pre-incubated with control TECs.

## Discussion

This work presents a detailed comparative phenotyping analysis of thymic and blood Treg in MG and pinpoints a potential role of TECs in the thymic immunoregulatory defects. The main results of the study are: (1) a significant difference in the phenotype of thymic CD4 cells compared to peripheral cells in MG patients. Patients’ thymic cells present alterations more striking than peripheral cells; (2) a significant impact of TECs is shown; (3) a potential participation of TSLP in the pathogenic processes is proposed and has never been described.

### Thymic and Peripheral Treg in Myasthenia Gravis

Published data are quite inconsistent for Treg numbers in MG. Several studies reported that percentages of Treg were unchanged in MG (10, 36, 37), while others reported some changes (38, 39). Previous studies investigated either cells extracted from the thymic tissue or blood, and the majority found that Treg from MG patients have impaired immunosuppressive functions in both tissues (10, 36, 40). This study is the first study to compare the function and phenotype of Treg from the inflammatory organ, the thymus, and from peripheral blood in control individuals and MG patients. We observed lower percentages of thymic, but not circulating, Treg in MG patients, regardless of whether we defined them as CD25hi or CD25 + CD127-. Treg fractioning based on CD45RA expression revealed that both rTreg and eTreg were reduced in the thymus, but not in the blood, of MG patients. These results suggest that thymic specific factors could have impacted the phenotype of thymic Treg in MG. Surprisingly, FoxP3 expression was unaltered in MG, neither in the thymus nor in the periphery, consistent with the publication by Kohler et al. (39), but not with that of Thirrupathi et al. (36). To explain these apparent contradictions, we propose several explanations: (1) even if some reports indicate changes in the numbers of Treg, it is a mild one that could be easily missed in other cohorts due to patients heterogeneity. Disease duration or corticosteroid treatment could also impact the number of Treg. In multiple sclerosis, while both circulating rTreg and eTreg have an impaired suppressive function in patients with a disease duration lower than 10 years, eTreg appeared to have a restored function in patients with a longer disease duration (41). It will be interesting to determine if the differences we observed are affected by the disease duration. (2) it is also possible that these differences are dependent on experimental conditions. For example, using fresh total cells, or purified frozen cells could lead to different results as the cell subsets analyzed are not strictly the same ones; in addition, the methods used (PCR versus flow cytometry) and the nature of the antibody clone could also induce differences in the results.

Altogether, our results indicate that percentages of thymic Treg are decreased in MG, confirm the immunosuppressive impairment of Treg in MG patients and show that thymic Treg are phenotypically different from the peripheral Treg. These results are in line with previous data showing that changes in the percentage of cells expressing high level of CD95 is much more pronounced in the thymus than in the periphery (8).

### What Could Be the Role of the Thymic Epithelium in Myasthenia Gravis?

We previously showed that the inflammatory environment in the MG thymus could play an important role in the pathogenesis of MG by generating T cells that are out of control (11), but the thymic component responsible for this impairment was not identified in this work. Previous publications including ours, demonstrated that the thymic epithelium in MG presents some functional alterations. TECs from MG patients produce excessive amounts of cytokines (IL-6 and IL-1β), chemokines (CXCL13 and RANTES), as well as kinases (p38 and ERK1/2 MAPKs) (15, 42– 44). We recently showed that MG TECs overproduce IL-23 and TGF-β3 (16). Together with the increase in IL-6, and IL-1β, MG-TECs provide a pro-inflammatory context that contributes to the differentiation and activation of pathogenic Th17 cells (16). In addition, IFN-β that is overexpressed in MG thymuses (13) increases the expression of the autoantigen (α-AChR) and the production of CXCL13 and BAFF by mTECs (14). The current study provides a straightforward evidence that the thymic epithelium plays a role in the functional alterations of Treg in MG. We were able to show that control thymic CD4 + T cells co-cultured with MG TECs lost a part of their ability to suppress Tconv proliferation.

Interestingly, the epithelium has also been implicated in other autoimmune pathologies, such as Sjogren’s syndrome (SS) and primary biliary cholangitis (PBC). In SS patients, the functional impairment of salivary glands comes as a result of an immune attack on epithelial cells of the affected organs, which has been named autoimmune epithelitis (45). Similar to MG, the epithelial cells overproduce cytokines and chemokines. Thus, we hypothesize that a defect in the epithelium mediates the pathogenesis in MG. How could this be possible? The epithelium is frequently an entry point for microorganisms and is constituted of one or several layers of epithelial tissue in many organs. The thymus is not an exception since a monolayer of epithelial cells surrounds each thymic lobule. In some autoimmune pathologies, the epithelial tissue undergoes increased apoptosis, which could lead to a presentation of many epithelial antigens. Although, this has not been shown in *ex vivo* experiments, indirect arguments favor such a possibility since IFN-β induces the TECs death *in vitro* and the uptake of TECs proteins by dendritic cells (14). The presence of viruses in the epithelium could lead to the production of IFN-I that will favor the recruitment of inflammatory cells and activate them to induce an immune response. Besides, the epithelial cells could play the role of antigen-presenting cells as they express HLA class II antigens and are capable of expressing co-stimulatory molecules.

In the current study, we showed that excessive TSLP production by MG TECs reduced the suppressive function of Treg.

The relationship between TSLP, Treg, and human thymus was described for the first time in 2005 by Watanabe et al. who showed that Hassall’s corpuscles induce the proliferation and differentiation of Tconv into Treg via the production of TSLP that activate thymic dendritic cells (46). Other findings show that TSLP plays an important role in the expansion and survival of CD4 + T cells (47). In addition, in the asthmatic allergy model, it was shown that TSLP directly and selectively impairs IL-10 production of Treg and inhibits their suppressive activity (48). Together these findings show that TSLP has a complex role and could either promote the differentiation of Treg or impairits production. The likely explanation is the existence of two variants for TSLP in human tissues. The main isoform (short form) is expressed in steady-state and plays a homeostatic role, while the long form is upregulated in inflammatory conditions (49). In our study, it is likely that the increased expression of TSLP by mTEC is the inflammatory long-form. Although we did not analyze IL-10 production, we showed that inhibition by the anti-TSLP antibody could restore the defective suppressive function of patients’ CD4 + T cells. Interestingly, viral stimuli could enhance TSLP expression by pulmonary epithelium (50); thus, the increased production of TSLP by MG TECs could be due to a viral stimulus in the thymus, a hypothesis previously discussed (4). In favor of this hypothesis, we showed that control TECs stimulated with Poly(I:C) or with IFN-I, increase their production of TSLP (data not shown). Interestingly, in human esophageal epithelial cells or in lung fibroblasts, the long isoform but not the short one is regulated by poly(I:C) or TNF-α, respectively (51, 52).

### SIgnificance of CD31 Decrease in MG CD4+ T Cell Subsets

The CD31 molecule, also called PECAM-1, is expressed on the surface of many cells, including cells of the immune system. In CD4 + T cells, CD31 is often regarded as a marker for naive cells, although some memory cells also express CD31. The expression of CD31 is lost after *in vitro* stimulation and expansion cycles (42). It, therefore, appears that CD31 is a differentiation antigen of CD4 + T cells, which is lost during maturation and proliferation. CD31 helps to control T-cell activation, and in its absence, T cells have a higher propensity to become activated, resulting in increased susceptibility to becoming apoptotic (43). Also, expression of PECAM-1 is important for dampening levels of multiple pro-inflammatory cytokines on the cellular and whole animal level (45).

Expression of CD31 is found on blood T cells that had recently emigrated from the thymus (53). This cell subset has an enhanced proliferative capacity, and it has been proposed that these cells enhance susceptibility to autoimmune disease (44). Our study revealed that in the thymus, rTreg have a decrease in CD31, suggesting that this particular population was probably activated and stimulated without acquiring the CD45RO marker memory marker. Interestingly in the periphery, CD31 is not altered in Treg, which underlines the difference with thymic Treg. However, CD31 expression is decreased in the overall CD4 population, indicating that CD31 is decreased in Tconv. The significance of this finding is probably increased activation and stimulation of these cells. These results show that although the Treg are not altered in the blood of MG patients, other pathogenic mechanisms might take place in the periphery. In a general context, the number of CD31 + cells was found to be diminished in several pathologies. Reduced CD31 + T cells is a hallmark of atherosclerotic plaque thrombosis (54). Multiple sclerosis is associated with lower percentages of circulating rTreg expressing CD31 (13).

In conclusion, this work highlights the role of the thymus in MG, as it shows for the first time that alterations in CD4 cells are more pronounced in the thymus than in the blood. We here explain the causes for thymic CD4 alterations; we demonstrate that TECs play a major role in Treg thymic defects and that TSLP overproduced by MG TECs is one of the soluble mediators that contributes to the thymic immune defects. Finally, replacing MG TECs by normal TECs allowed restoring the immunosuppressive abilities of MG Treg. These findings demonstrate that in a favorable environment, it is possible to reestablish the regulatory function of Treg in MG patients.

## Data Availability Statement

The datasets generated for this study are available on request to the corresponding author.

## Ethics Statement

The studies involving human participants were reviewed and approved by Local Ethics Committee (“Comité Consultatif de Protection des Personnes”), Ile de France VII (Kremlin Bicêtre, France). The relevant authorization numbers are ID RCB 2006-A00164-47 and 2010-A00250-39. Written informed consent to participate in this study was provided by the participants’ legal guardian/next of kin.

## Author Contributions

FT, DN, and AG performed and analyzed the experiments. FT collected the samples. BE provided the blood samples. EF and RR provided the thymic samples. JV, RL, and SB-A read and revised the manuscript. FT, JV, and SB-A analyzed the experiments and wrote the manuscript.

## Conflict of Interest

The authors declare that the research was conducted in the absence of any commercial or financial relationships that could be construed as a potential conflict of interest.
