# Supplementary material for: Comparative Analysis of Thymic and Blood Treg in Myasthenia Gravis: Thymic Epithelial Cells Contribute to Thymic Immunoregulatory Defects
Source: Front Immunol. 2020 May 6;11:782. doi: 10.3389/fimmu.2020.00782 (PMC7218102; doi:10.3389/fimmu.2020.00782)
Supplement: Supplementary file 1 [file Presentation_1.pptx]

## Slide 1
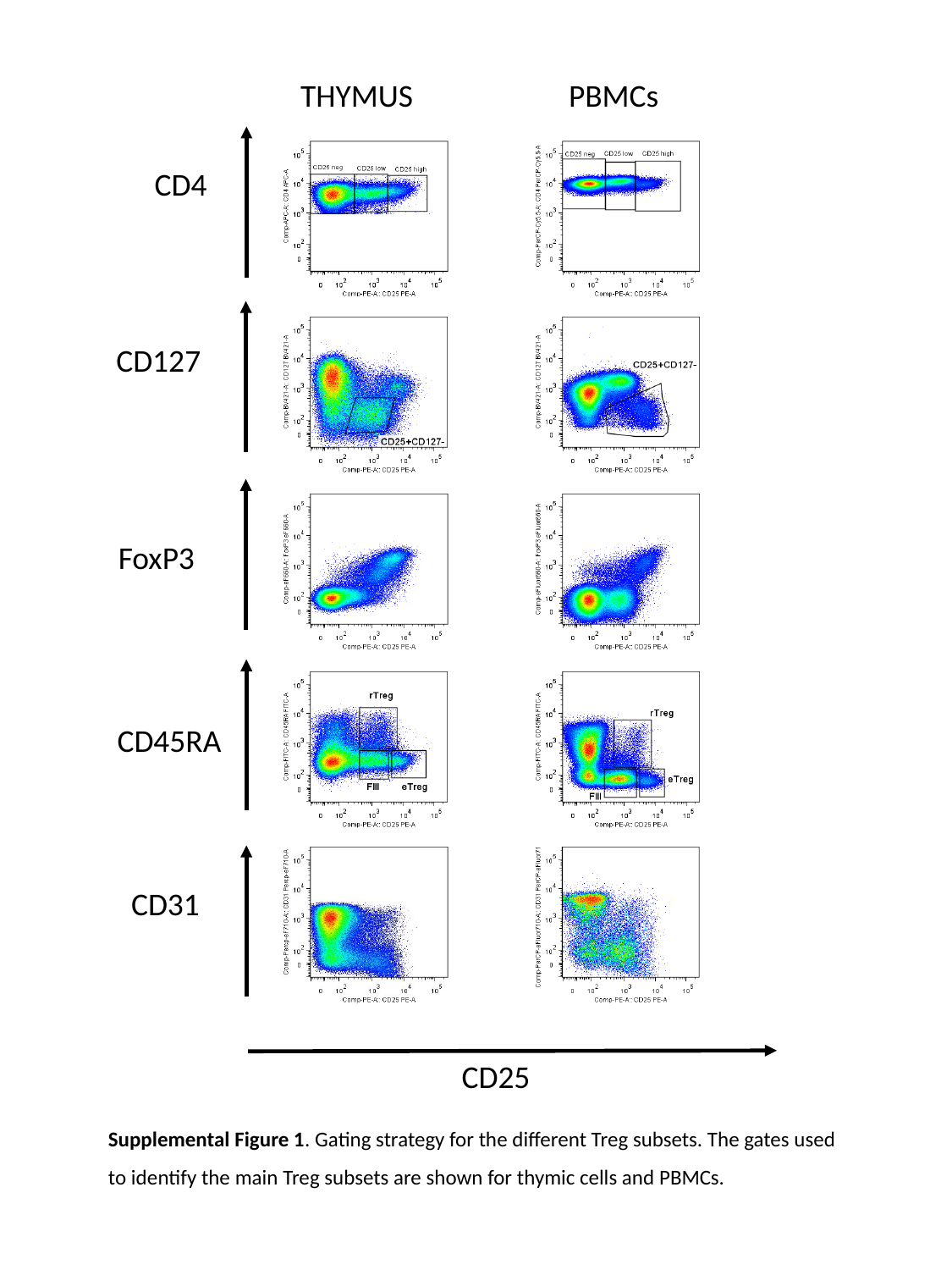

THYMUS
PBMCs
CD4
CD127
FoxP3
CD45RA
CD31
CD25
Supplemental Figure 1. Gating strategy for the different Treg subsets. The gates used to identify the main Treg subsets are shown for thymic cells and PBMCs.

## Slide 2
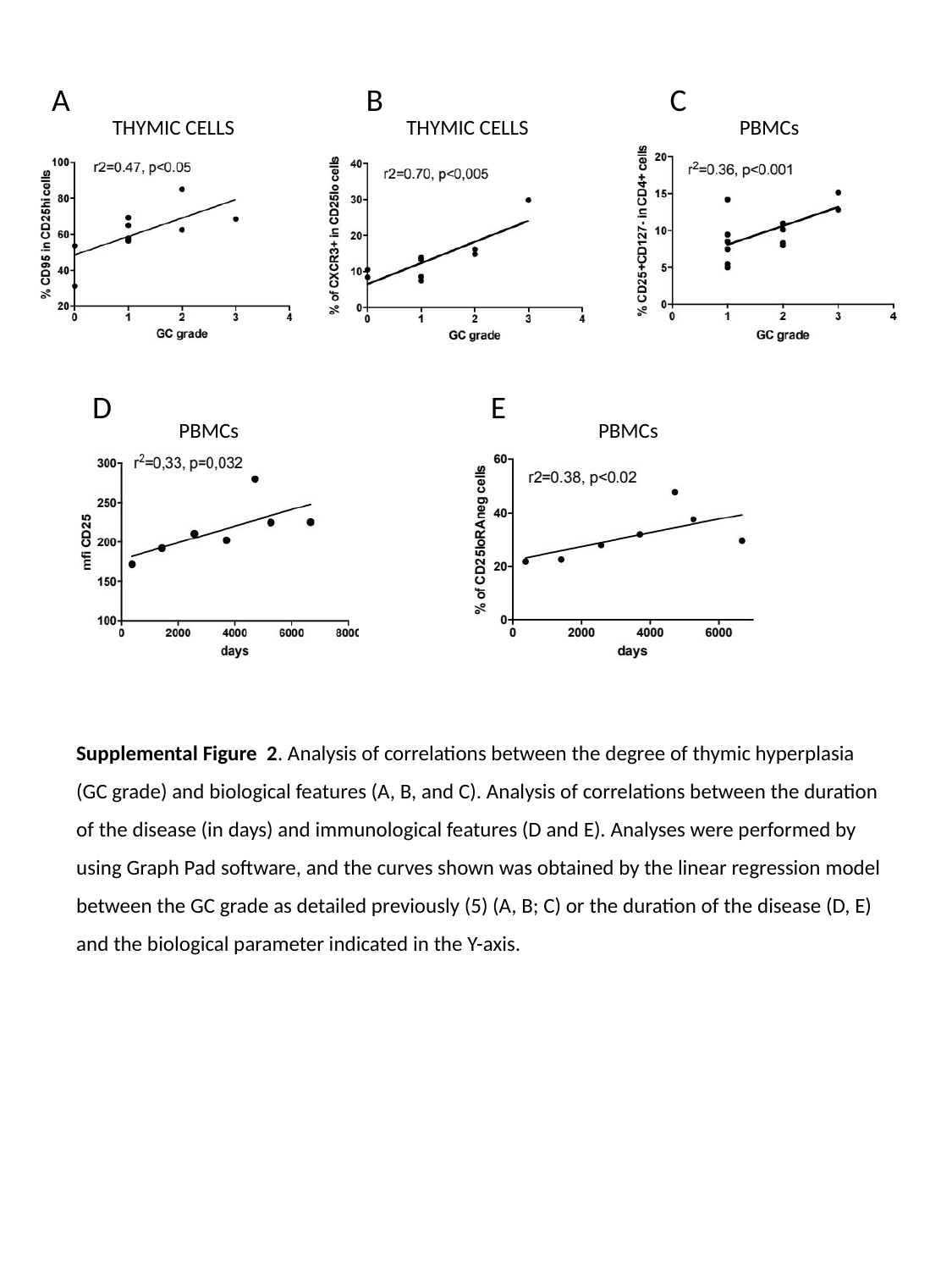

A
B
C
THYMIC CELLS
THYMIC CELLS
PBMCs
D
E
PBMCs
PBMCs
Supplemental Figure 2. Analysis of correlations between the degree of thymic hyperplasia (GC grade) and biological features (A, B, and C). Analysis of correlations between the duration of the disease (in days) and immunological features (D and E). Analyses were performed by using Graph Pad software, and the curves shown was obtained by the linear regression model between the GC grade as detailed previously (5) (A, B; C) or the duration of the disease (D, E) and the biological parameter indicated in the Y-axis.

## Slide 3
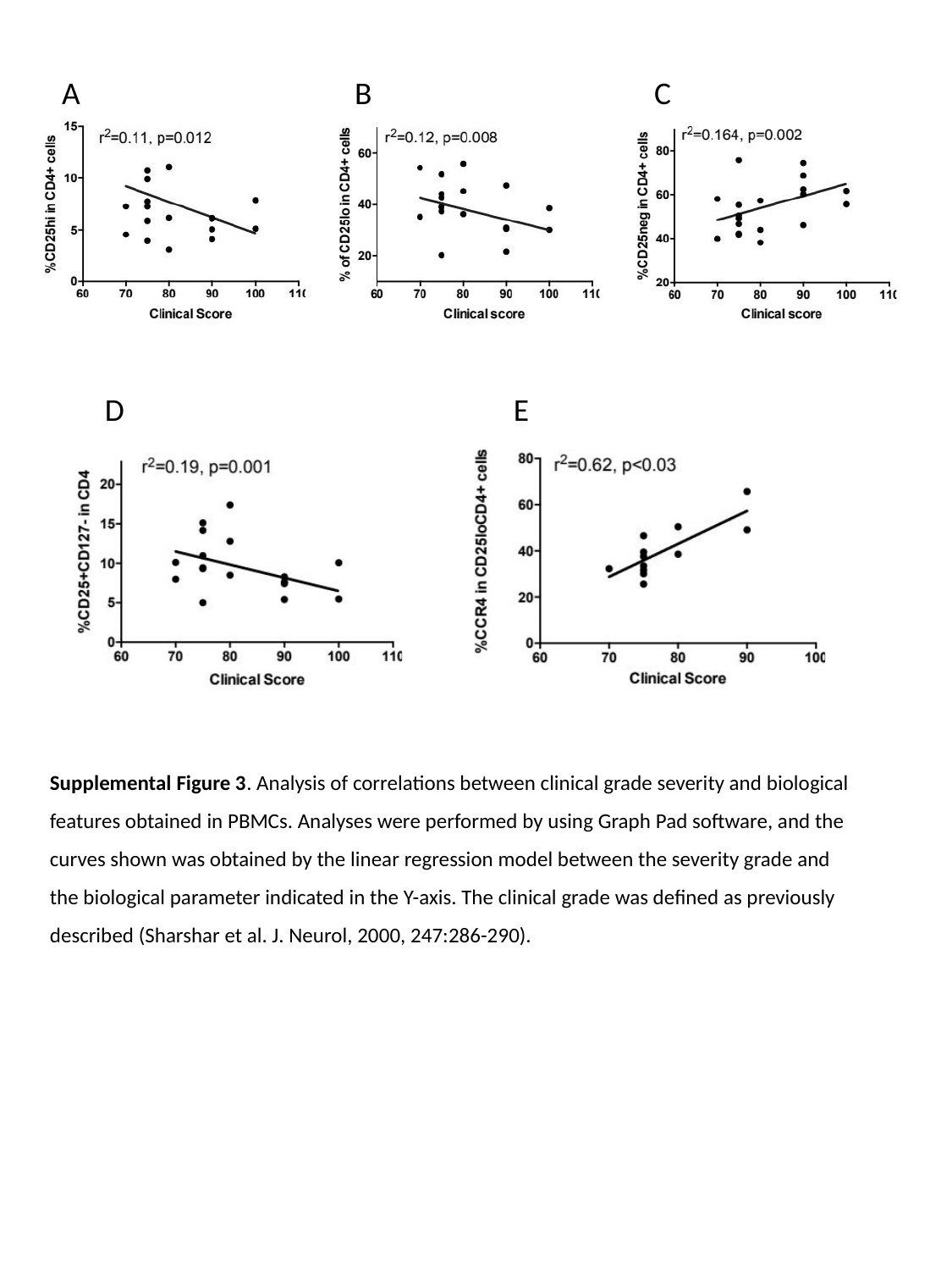

A
B
C
D
E
Supplemental Figure 3. Analysis of correlations between clinical grade severity and biological features obtained in PBMCs. Analyses were performed by using Graph Pad software, and the curves shown was obtained by the linear regression model between the severity grade and the biological parameter indicated in the Y-axis. The clinical grade was defined as previously described (Sharshar et al. J. Neurol, 2000, 247:286-290).

## Slide 4
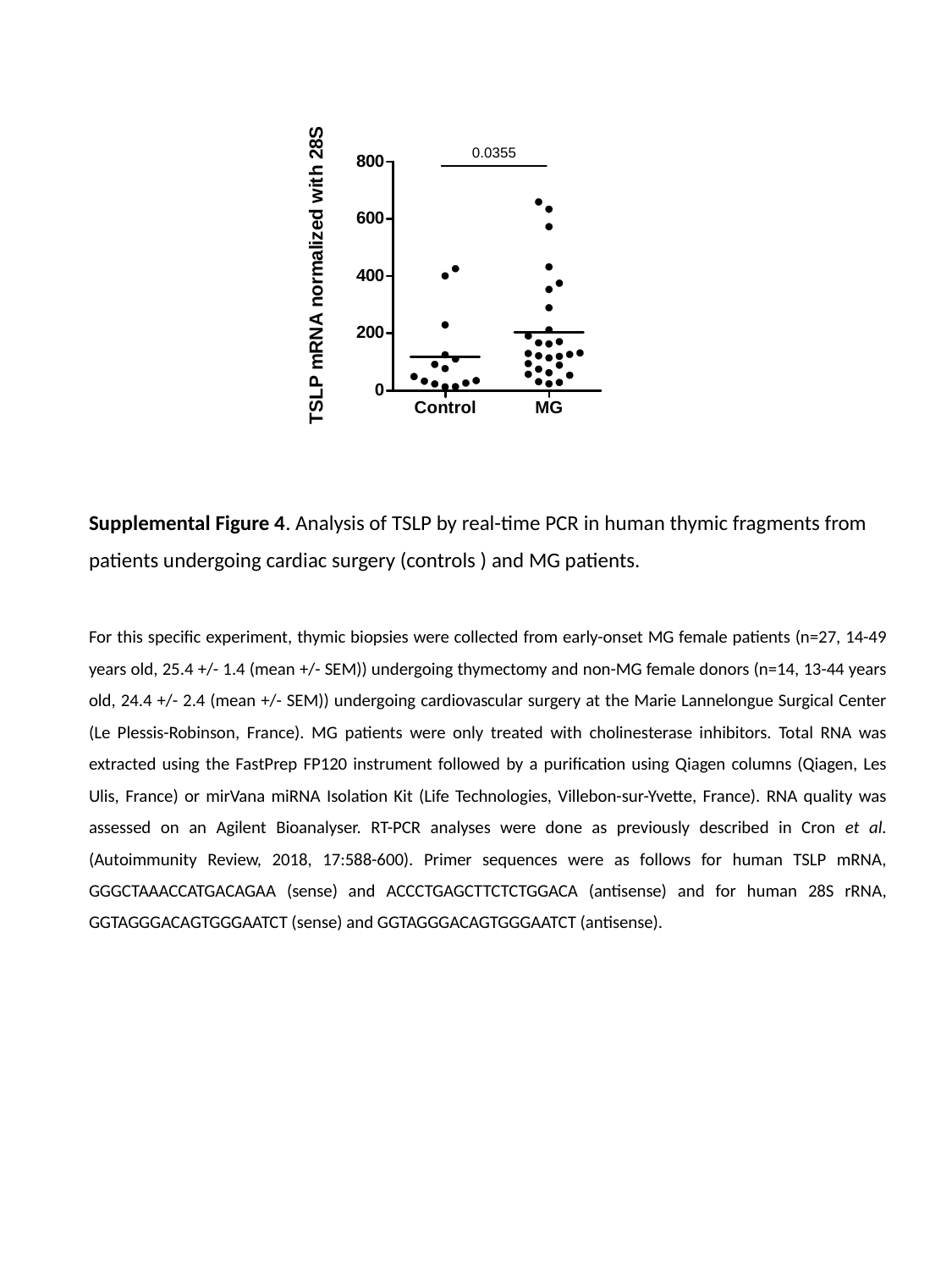

Supplemental Figure 4. Analysis of TSLP by real-time PCR in human thymic fragments from patients undergoing cardiac surgery (controls ) and MG patients.
For this specific experiment, thymic biopsies were collected from early-onset MG female patients (n=27, 14-49 years old, 25.4 +/- 1.4 (mean +/- SEM)) undergoing thymectomy and non-MG female donors (n=14, 13-44 years old, 24.4 +/- 2.4 (mean +/- SEM)) undergoing cardiovascular surgery at the Marie Lannelongue Surgical Center (Le Plessis-Robinson, France). MG patients were only treated with cholinesterase inhibitors. Total RNA was extracted using the FastPrep FP120 instrument followed by a purification using Qiagen columns (Qiagen, Les Ulis, France) or mirVana miRNA Isolation Kit (Life Technologies, Villebon-sur-Yvette, France). RNA quality was assessed on an Agilent Bioanalyser. RT-PCR analyses were done as previously described in Cron et al. (Autoimmunity Review, 2018, 17:588-600). Primer sequences were as follows for human TSLP mRNA, GGGCTAAACCATGACAGAA (sense) and ACCCTGAGCTTCTCTGGACA (antisense) and for human 28S rRNA, GGTAGGGACAGTGGGAATCT (sense) and GGTAGGGACAGTGGGAATCT (antisense).
